# Stochastic Theory of Early Viral Infection: Continuous versus Burst Production of Virions

**DOI:** 10.1371/journal.pcbi.1001058

**Published:** 2011-02-03

**Authors:** John E. Pearson, Paul Krapivsky, Alan S. Perelson

**Affiliations:** 1 Theoretical Biology & Biophysics, Los Alamos National Laboratory, Los Alamos, New Mexico, United States of America; 2 Department of Physics, Boston University, Boston, Massachusetts, United States of America; Imperial College London, United Kingdom

## Abstract

Viral production from infected cells can occur continuously or in a burst that generally kills the cell. For HIV infection, both modes of production have been suggested. Standard viral dynamic models formulated as sets of ordinary differential equations can not distinguish between these two modes of viral production, as the predicted dynamics is identical as long as infected cells produce the same total number of virions over their lifespan. Here we show that in stochastic models of viral infection the two modes of viral production yield different early term dynamics. Further, we analytically determine the probability that infections initiated with any number of virions and infected cells reach extinction, the state when both the population of virions and infected cells vanish, and show this too has different solutions for continuous and burst production. We also compute the distributions of times to establish infection as well as the distribution of times to extinction starting from both a single virion as well as from a single infected cell for both modes of virion production.

## Author Summary

The dynamics of HIV infection and treatment has been extensively studied using ordinary differential equation models. Recent work on HIV transmission has suggested that most sexually transmitted infections are started by a single virus or infected cell. This observation coupled with the fact that successful HIV transmission only occurs in 1 per 100 to 1 per 1000 coital acts suggests that early events in infection are stochastic. Here we develop a stochastic model of HIV infection and use it to characterize the dynamics of early infection when virus is released from cells either continuously or in a burst. We show that these mechanisms of viral production produce different early dynamics, with different probabilities of extinction and different distributions of time to establish infection. In deterministic models, these modes of viral production are indistinguishable.

## Introduction

The earliest events in infection are stochastic. Whether exposure to virus leads to systemic infection or complete elimination of the virus can be a matter of luck, particularly when exposure is to low levels of virus. For example, the transmission probability for HIV infection is 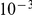
–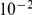 per coital act [1]–[3]. In 80% of HIV infections of heterosexuals, a single viral strain is transmitted or founds the infection [4]. In most cases after sexual exposure to HIV, infection fails to take off. When it does take off it likely does so from a single infectious virion or a single infected cell. Whether exposure to virus, be it HIV or the common cold, results in persistent infection or elimination hinges on numerous poorly understood factors including antibody and innate immune responses, virus specific cytotoxic T lymphocyte responses, the spatial distributions of these components [5], and pure chance.

Here we study some simple viral infection models in a stochastic setting using HIV as a model system. The models that we consider are relevant for the earliest stages of infection before target cells are depleted to any extent and before immune responses are stimulated. Thus, we consider models with no immune response in which the number of target cells, 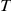, is held fixed and where we consider only variations in the number of virions, 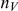, and the number of infected cells, 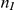, with 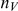 and 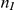 being non-negative integers. We derive exact analytic expressions for the *extinction* probability, i.e., the probability that the virus and all infected cells are completely eliminated from the host, for two related models that differ in the manner in which virus is produced. We also present simulation results for the conditional mean time to observable infection.

The extinction problem is related to the classic “gambler's ruin” problem [6], which Pascal [7] first solved and then posed to Fermat, hoping in vain to stump him, and to Huygens [8] who thought there might be some applicability to disease and wrote “For what can there be in common between the Value of a Chance in a Game, and the Knowledge and Cure of a Distemper? And how can the nicest Determination of the former, any way influence or illustrate the latter?” More recently Tan and Wu [9] developed a 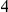-dimensional stochastic infection model for HIV that incorporated target cells and latently infected cells and studied it via Monte Carlo simulations. They noted that there was positive probability that the virus could be eliminated by the process [9]. Monte Carlo approaches were also used by Kamina et al. [10] and Heffernan and Wahl [11] to study the probability that an infection would not become established after exposure to a viral inoculum of a given size. Tuckwell and Corfec [12], [13] developed similar multi-dimensional models to study early infection but modeled them as diffusion processes via simulation of stochastic differential equations. Merrill [14] modeled early infection as a branching process that kept track of the number of infected cells but not of virions. Lee et al. [15] also modeled only infected cell dynamics during acute infection but focused on the stochastic changes in HIV genetic sequences starting from an infection initiated by a single HIV genome. Tuckwell et al. [16] studied the probability of viruses entering a host infecting one or more target cells before being cleared, but did not carry out a detailed analysis including infected cells. Haeno and Iwasa [17] developed a stochastic model of early infection in order to study the generation of drug resistant virus in an exponentially expanding viral population. Ribeiro and Bonhoeffer [18] also develop a stochastic simulation of early infection in which only infected cells are follwed to study the best time to start antiretroviral therapy in a model with stochastic generation of drug resistant mutants. In this manuscript we model early infection as a discrete random process in which both the number of virions and the number of infected cells are followed.

The form of the models that we develop are similar to those used in epidemiology to study the spread of infection from person to person [19]. As such we will find that the basic reproductive number, 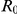, first introduced in epidemiology to denote the average number of people infected by one infected person put into a population of susceptibles, plays a role in our analysis. Here 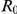 will denote the average number of new cells infected by one cell during its lifetime when placed in a population of fully susceptible cells. As in epidemiology, we will find that when 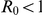 infections will surely die out and when 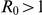 there is a positive probability that the infection will die out. Our goal here is not simply to reiterate these well known results but rather to uncover basic features of early HIV infection and to study the differences between continuous and burst viral release.

## Model

One of the simplest infection models consists of virions, (V), target cells (T), and productively infected cells (I) with transitions [20]:
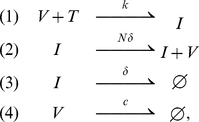
(1)where 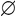 denotes the empty set and indicates that infected cells or virus is being cleared. The symbols above the arrows denote the rates of the various processes, where 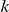 is the rate constant characterizing infection, 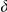 is the death rate of infected cells, 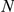 is the viral burst size, i.e., the total number of virions produced by an infected cell over its lifetime, 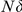 is the rate at which infected cells produce virus, and 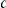 is the virion clearance rate [20]. In some models, particularly those in which a cytolytic lymphocyte response may affect lymphocyte lifespan, the symbol 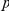 is used to denote the virion production rate rather than 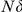 [20]. Here, where we focus on the earliest events in infection, before there is an immune response, using 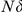 for the virion production rate allows us to simplify some expressions. Also, because we are focusing on *early* infection we neglect variations in the number of target cells. This is justified because, as we show below, only a tiny fraction of target cells need be infected to insure that the infection will persist. Thus the model above can be written:
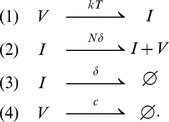
(2)We call the model specified by Eq. (2) the “continuous production” model because once a cell is infected it produces virus continuously throughout its life.

A slightly different but related model is given by the set of transitions
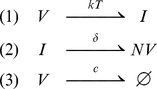
(3)We call the model specified by Eq. (3) the “burst” model because once a cell is infected it releases all its virus in a single burst simultaneous with its death. Although an infected cell may not burst as in a lytic phage infection of bacteria, HIV may be rapidly produced towards the end of an infected cell's lifespan as in other retroviral infections [21]. Also, because we are studying very early infection, before immune responses begin, we assume death of a cell is due solely to viral cytopathic effects and hence ignore the possibility that death occurs before 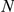 virions are released.

Both models have identical mean-field kinetics given by:
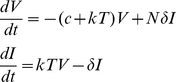
(4)where 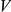 and 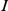 are the concentrations of virus and infected cells. At the deterministic level the burst and continuous production models make the same predictions. Note that this model differs from the “standard” model of viral infection in that viral clearance occurs at rate 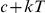 rather than at rate 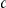, i.e., the model keeps track of the fact that one virus is lost every time a cell is infected. However, since 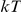 is a constant the model is equivalent to the standard model in which 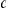 in the standard model incorporates virion loss due to infection [22].

Note that the origin (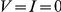) is a steady state of the deterministic system. The origin is a stable steady state provided the basic reproductive ratio 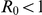, where 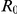 is the number of new cells infected by an infected cell during its lifetime with
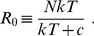
(5)Although this is easily seen by calculating the determinant of the linear system specified in Eq. (4) it is worth noting that for HIV, (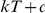) is large compared to 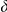 and virions become “slaved” to infectious cells [23], so that 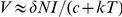, which results in 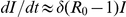. We show that if 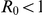 virus and all infected cells will be eliminated with certainty. Unlike deterministic models, for 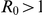 there is still a finite probability that the virus and all infected cells will be eliminated stochastically. We shall also see that once the virus “takes off”, it roughly satisfies the slaving approximation 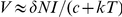, while before it takes off the dynamics are fundamentally stochastic.

## Results

### Stochastic Extinction

We consider systems which can be fully specified by a state vector 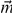. For both the burst and continuous production models 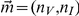, where 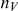 and 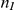 are the number of virions and infected cells, respectively. Upon a transition the state 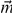 is incremented by one of the transition vectors 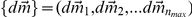 where 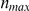 is the maximum number of transitions the system can make out of any state. For the continuous production model we have 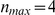 and 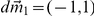, 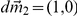, 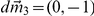, and 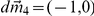. The rate of the 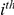 reaction is given by 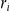. Thus, for the continuous production model there are four types of reactions: (1) infection with rate 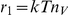, (2) viral production with rate 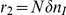, (3) death of a infected cell with rate 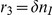, and (4) virion clearance with rate 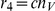. The probability that the 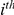 reaction is the next reaction is given by Gillespie's algorithm [24]:
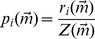
(6)where
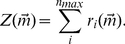
(7)For the continuous production model, 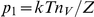, 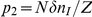, 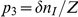, 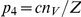, and 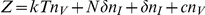. The time of the next reaction is a random variable with distribution 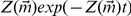. For the burst model, 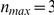, 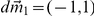, 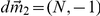, 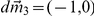 and the corresponding reaction rates are 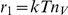, 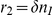, and 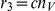.

Our goal is to determine the probability that an exposure to virus eventually evolves to “extinction”, i.e., 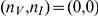. Throughout this article, we refer to the loss of all virus and infected cells from the host as “extinction” and to the decay of virus as “clearance”.

**Figure 1:**
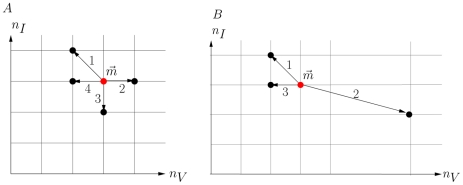
State space diagrams. (A) Continuous production model. Starting from the state 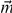 (the red dot) there are four possible reactions from the point 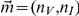 to the neighboring points. As stated in the text the possible transitions are 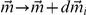 where 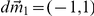, 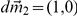, 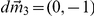, 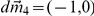. (B) Burst Model. Starting from the state 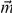 (the red dot) there are three possible reactions from the point 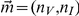 to the neighboring points. As stated in the text the possible transitions are 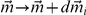 where 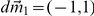, 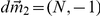, 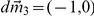. For both models the 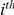 reaction occurs at rate 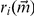 with probability 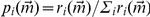 as discussed in the text.

Stochastic extinction is a multi-dimensional analogue to the classic gambler's ruin problem first solved by Pascal [7]. The extinction probability, 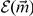 from state 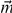, satisfies [6], [25]–[27]:
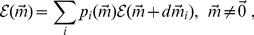
(8)

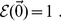
(9)Equation (8) can be understood from Figure 1. If the system starts out in state 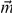 on the first transition the state will jump to one of the 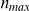 states 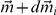, 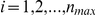, with probability 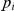. Clearly, then the extinction probability from state 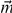 is the weighted sum of the extinction probabilities at the neighboring sites where the weights are just the probabilities of making the individual transitions. Note that 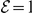 is always a solution since 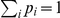.

Although the general solution is intractable we will show that if processes of virion and infected cell extinction are independent, the functional equation for 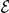 can be reduced to an algebraic one. Since each virus and infected cell acts independently in our model, we assume:
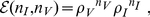
(10)where 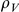 and 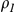 are the probabilities that a process initiated with a single virion or single infected cell, respectively, results in extinction. Using Eq. (10), Eqs. (8–9) can be reduced to algebraic equations for 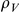 and 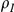. In the following we carry out this program for both the continuous and burst models.

### Extinction Probability for the Burst and Continuous Production Models

For the continuous production model, substituting Eq. (10) into Eqs. (8–9) yields
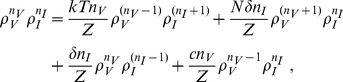
(11)where 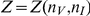. We convert this system of equations to a pair of algebraic equations by first setting 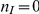 and obtaining a first equation and then setting 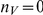 to obtain another. Note that 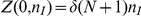 and 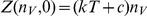. Thus we obtain the pair of equations
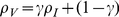
(12)

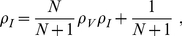
(13)where 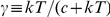 is the probability that a virion infects a cell. Note from the definition of 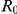, 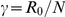. Substituting Eq. (12) into Eq. (13), we obtain a quadratic equation with solutions, 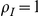 and 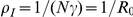. Substituting into Eq. (12), we find 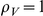 and 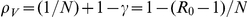. Since probabilities need to be less than or equal to 1,
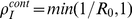
(14)

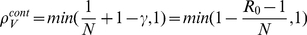
(15)for the continuous production model single cell and single virion extinction probabilities. Thus, if 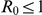, 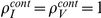, whereas if 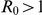, 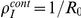 and 
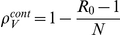
.

For the burst model, as we show in the next section, a similar analysis yields
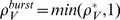
(16)

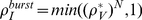
(17)where 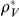 is a positive real root of
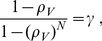
(18)or equivalently of
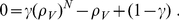
(19)Noting that 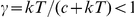 for 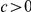 and using Descartes' rule of signs shows that there are either 2 or 0 real positive roots. Since 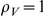 is one root, there is exactly one other positive root of Eq. 18, which we denote 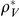. Note that if 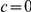 then 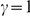 and there is only one root, 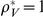.

**Figure 2:**
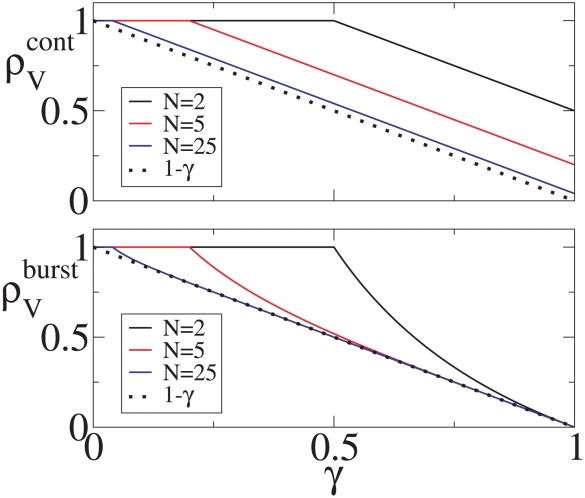
The single virion extinction probability, 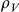, versus 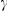, the probability that a virion infects a cell rather than is cleared. Dashed lines: burst model. Solid lines: continuous production model. Black dashed (solid) lines: 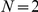 burst (continuous). Red dashed (solid) lines: 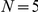 burst (continuous). Blue dashed (solid) lines: 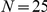 burst (continuous). The heavy dotted line is the limiting curve 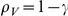.

Figure 2 shows the single virion extinction probability, 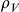, as a function of 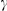 for 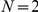, 5, and 25 for both the burst and continuous models. For large 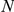 the extinction probabilities for both models converge to the diagonal line (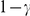) connecting the upper left to the bottom right corners. For both models the single virion extinction probability, 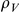, is a function of 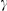 and 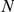 and that 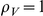 for 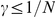, i.e, for 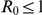. Also in both models if 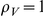 then 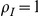 and in both cases extinction is certain if 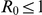. This is not a new result and could be derived from a branching process approach where the process would be subcritical if 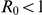 and then extinction would be guaranteed. Results along this line in the context of epidemiological models are summarized in Britton and Lindenstrand [28] and Britton [29]. Britton [29] also points out that Reed and Frost in a series of unpublished lectures from 1928 study an epidemiological model where all infections are assumed to occur exactly at the end of the infectious period, which is analogous to the burst model where infection can only be transmitted from one cell to another at the end of the infected cell's life.

The main difference between the two models is that 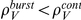. The difference between the two models is most easily understood in the 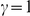 limit where the probability of a virion infecting a cell rather than being cleared approaches 1. Note that in the burst model 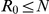 since the number of cells infected by a single infected cell must be less than or equal to the number of virions produced, and in the continuous production model this is true for the mean. As 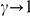 we find that 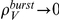 and 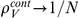. In the 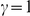 limit virus is not cleared in either model but disappears only by infecting another cell. In the burst model all infected cells result in the creation of 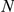 new virions. Thus, for the burst model, the extinction probability approaches zero as 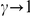. By contrast, for the continuous model there is a chance that an infected cell will die before it produces any virus.

In the infinite 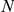 limit the single virion extinction probabilities become equal for the two models, i.e., 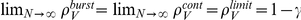. We have been focusing on the single virion extinction probability 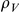. Note that 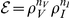 and that for 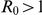, 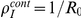, and 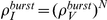. In the large 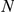 limit if 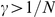 then 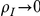 for both models. Thus in the large 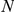 limit the probability of stochastically clearing the infection is effectively zero if any cells at all are infected, since each infected cell is assumed to produce an arbitrarily large amount of virus in this limit.

#### Random burst size

In the burst model considered above, every infected cell releases 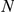 virions, the notion being that a cell produces virus until a critical number 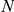 is reached, at which time it releases the entire stock of virus that it has produced since infection. Here we consider a generalization of the burst model, in which the burst size is a random variable so that the probability of a burst of size 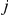 is 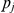, with 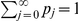. In this case, reaction 2 in Eq. (3) becomes a set of reactions:
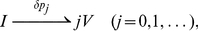
(20)where 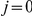 implies an infected cell dies before releasing any virus. For the generalized burst model, we use an analysis similar to the one above.

Substituting Eq. (10) into Eqs. (8–9) yields
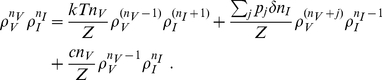
(21)We again convert this system of equations to a pair of algebraic equations by first setting 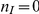 and obtaining a first equation and then setting 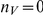 to obtain another. Note that 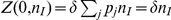 and that 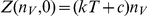. Thus we obtain the pair of equations
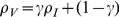
(22)

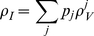
(23)with solutions
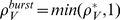
(24)

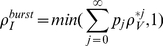
(25)where 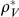 is a positive real root of
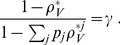
(26)As in the case of the burst model, there are two real roots when 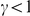, with one being 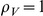 and the other denoted 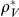. If 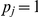 for 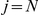 and 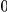 otherwise we have the burst model discussed in the previous section.

We investigated the single virion extinction probability, 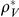 as a function of 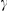 for 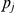 Poisson distributed with mean 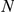 and compared it to the burst model (with 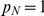 and 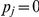 for 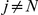). We found that the single virion extinction probability, 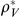, was similar for the random burst and burst models for 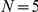 and nearly identical for 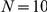.

### Dynamics

**Figure 3:**
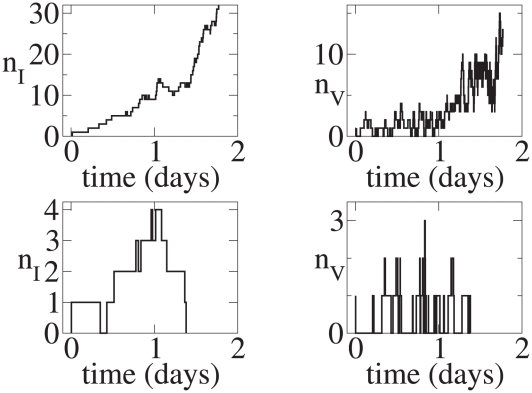
Continuous production model time series. Initial conditions: 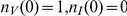. Top left: 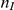 versus 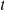 for a realization that leads to infection. Top right: 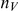 versus 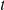 for the same realization. Bottom left: 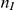 versus 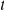 for a realization that leads to extinction. Bottom right: 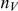 versus 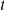 for the same realization. 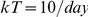, 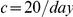, and 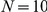.

**Figure 4:**
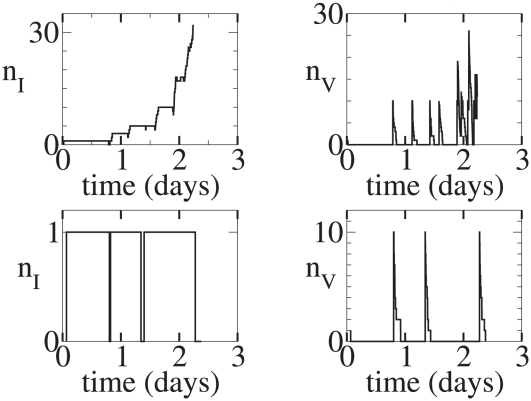
Burst model time series. Initial conditions: 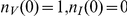. Top left: 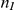 versus 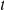 for a realization that leads to infection. Top right: 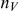 versus 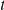 for the same realization. Bottom left: 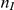 versus 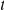 for a realization that leads to extinction. Bottom right: 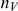 versus 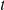 for the same realization. 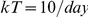, 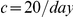, and 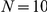.

**Figure 5:**
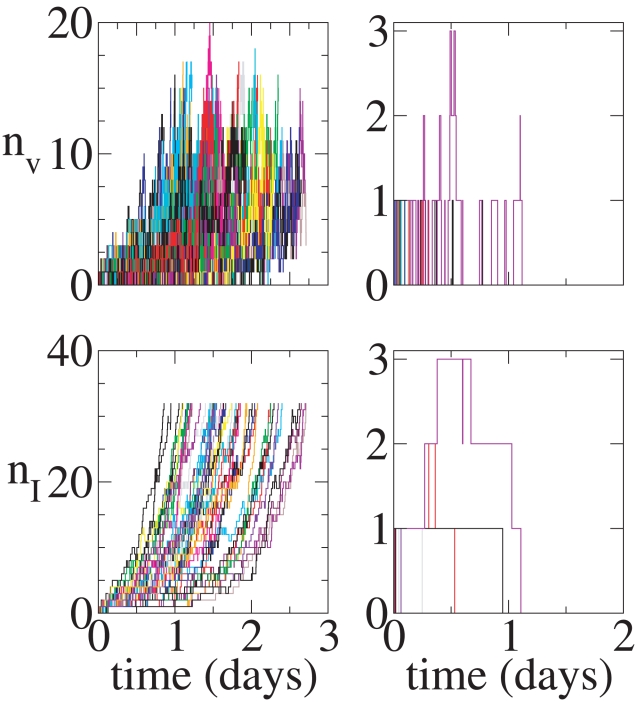
Continuous model. Representative time series. Initial condition: 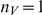, 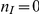. Left column: 100 realizations that lead to infection. Right Column: 100 realizations that lead to extinction.

**Figure 6:**
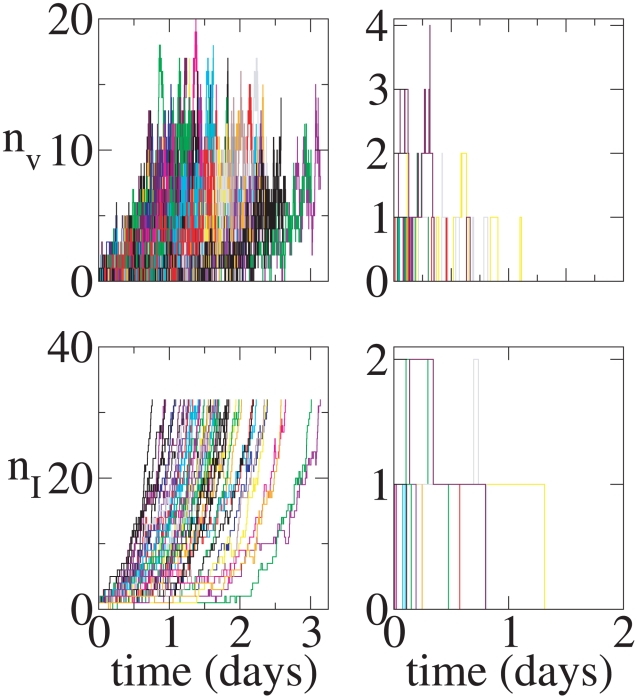
Continuous model. Representative time series. Initial condition: 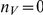, 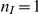. Left column: 100 realizations that lead to infection. Right Column: 100 realizations that lead to extinction.

**Figure 7:**
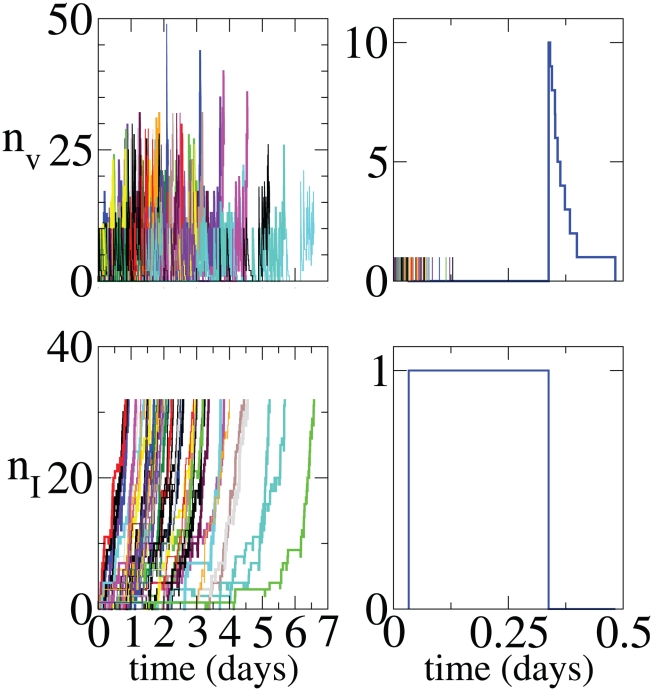
Burst model. Representative time series. Initial condition: 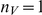, 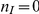. Left column: 100 realizations that lead to infection. Right Column: 100 realizations that lead to extinction.

**Figure 8:**
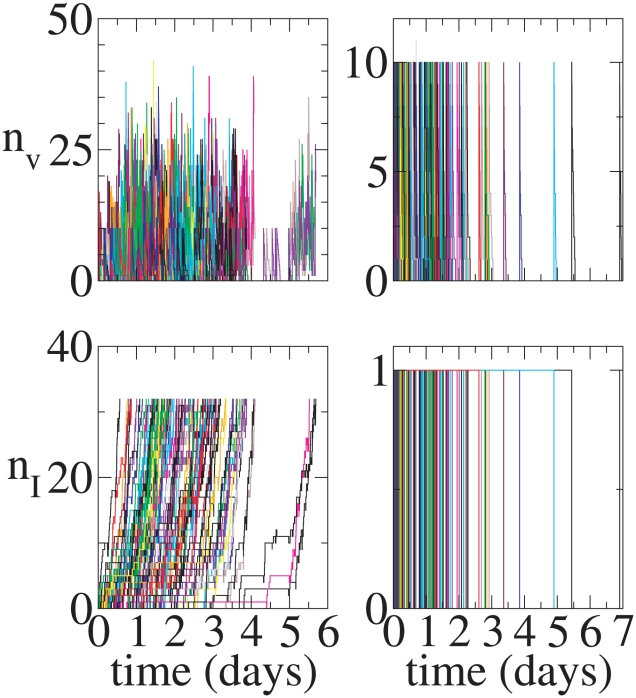
Burst model. Representative time series. Initial condition: 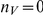, 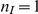. Left column: 100 realizations that lead to infection. Right Column: 100 realizations that lead to extinction.

**Figure 9:**
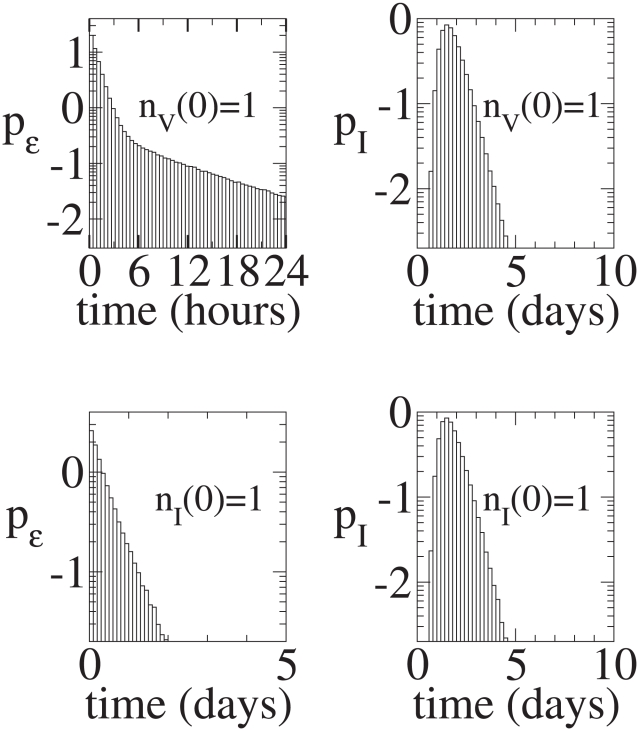
Continuous production model. Top Left: Distribution of times until an infection begun with a single virion goes extinct. The conditional mean time to extinction is about 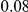 days for this parameter set. Top Right: Distribution of times until an infection begun with a single virion results in 32 infected cells, given that the infection does not go extinct. The conditional mean time until there are 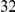 infected cells is 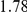 days. Bottom Left: Distribution of times until an infection begun with a single infected cell goes extinct. The conditional mean time to extinction is about 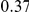 days. Bottom Right: Distribution of times until an infection begun with a single infected cell results in 32 infected cells, given that the infection does not go extinct. The conditional mean time until there are 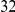 infected cells is 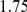 days.

**Figure 10:**
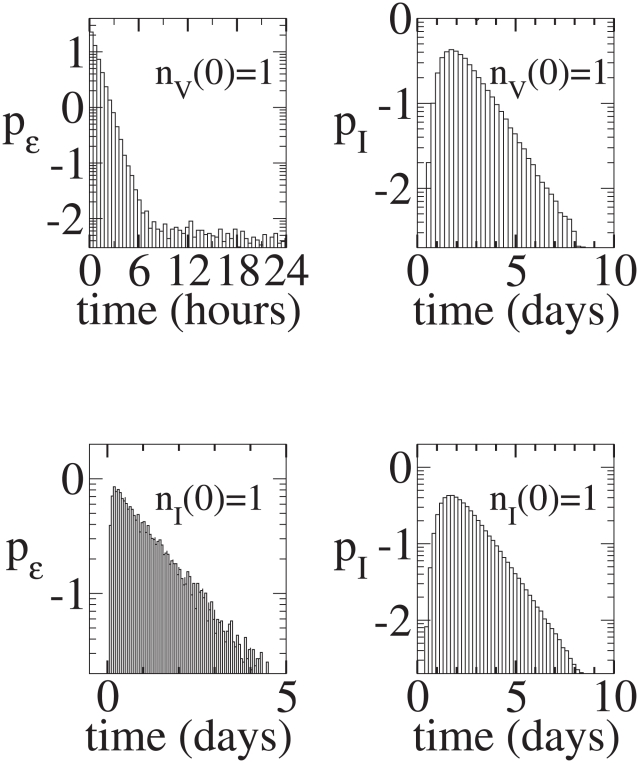
Burst model. Top Left: Conditional Distribution of times until an infection begun with a single virion goes extinct. The conditional mean time to extinction is about 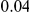 days. Top Right: Distribution of times until an infection begun with a single virion results in 32 infected cells, given that the infection does not go extinct. The conditional mean time until there are 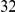 infected cells is 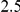 days. Bottom Left: Distribution of times until an infection begun with a single infected cell goes extinct. The conditional mean time to extinction is about 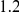 days. Bottom Right: Distribution of times until an infection begun with a single infected cell results in 32 infected cells, given that the infection does not go extinct. The conditional mean time until there are 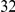 infected cells is 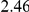 days.

The earliest stages of HIV and SIV infection have a characteristic “eclipse” phase during which the virus remains below the limit of detectability of current assays. Here we explore the role stochastic effects play in determining the length of the eclipse phase. Using Gillespie's stochastic simulation method [24] we compute the mean time to detectability following a one virion challenge. In Figures 3–10 we use the following parameters for illustrative purposes: 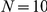, 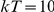/day, 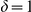/day [30], and 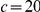/day [31]. For these parameter values, 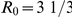, which is lower than the median value of 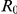 found by Ribeiro et al. [32], Stafford et al. [33] and Little et al. [34] during primary HIV infection. However, these estimates relied on data obtained after the virus was observable and in the case of Stafford et al and Little et al. mainly after the viral load peak. At earlier stages of infection, 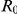 could be different.

A value of 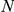 of about 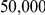 has recently been estimated for SIV infection in rhesus macaques [35]. However, not all virions are infectious. In the formulation given above we have assumed all virions are equivalent and hence equally infectious. Although one could generalize the model to include both infectious and noninfectious virions, following only infectious virions has the advantage of allowing one to track smaller numbers of virions in simulations. For virus isolated during chronic infection, approximately one in 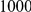 to 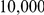 virions appear to be infectious [36]–[39], suggesting that if we model only infectious virus values of 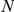 between 5 and 50 might be reasonable. As our default, we have chosen a value of 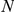 consistent with these estimates. Recent work has suggested that virus isolated early in infection has a higher ratio of infectious to noninfectious virus [40], and thus depending on the source of infecting virus larger values of 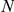 might be appropriate.

Our choices of default values of 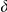 and 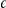 are based on estimates derived from data obtained during chronic infection [30], [31], and thus they too might not be appropriate for the earliest stages of infection. Lastly, the value of 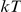 was chosen so that with the other parameter choices a sensible value for 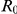 was obtained. Thus, while the parameter choices studied here are reasonable guesses based on what we know about HIV infection dynamics, there is some uncertainty about them.

Figures 3 and 4 show 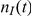 and 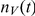 for the continuous production and burst models, respectively. As expected, in both cases infection can persist (top panels in Figures 3 and 4) or go extinct (bottom panels in Figures 3 and 4). Here we have arbitrarily defined persistent infection as 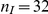. Although it is mathematically possible for the virus to be cleared by chance with 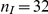, at this point the probability of stochastic extinction is on the order of 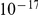. This is because for 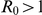, 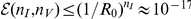. Also, virus becomes detectable in plasma with conventional assays when its concentration is 50 copies/ml. Assuming that deterministic equations are appropriate at this point, one finds that if virus and infected cells are at quasi-steady state then 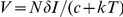. Thus, if each infected cell produces 50,000 virions [35], lives about a day while productively infected [30] and has a clearance rate (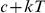) of about 23/day [31], then when 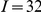, 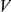 will be approximately 50 copies/ml assuming virus distributes through approximately 1.5 liters of extracellular body water in a 7 kg macaque. Thus, by the time 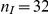 the eclipse phase of SIV infection should be over. For HIV infection the volume of distribution is about 10-fold larger (a 70 kg human has about 15 liters of extracellular body water) and thus virus detectability would be delayed until 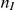 is about 10-fold larger. Nonetheless, the probability of extinction would still remain 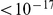.

In the realization that leads to persistent infection in the continuous production model, the initial virus quickly infects a single cell and that cell starts producing new virions. Thus, 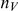 begins fluctuating from time zero as virions are produced and cleared stochastically (Figure 3). Further, these released virions infect new cells and 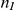 rises substantially over the first 2 days of infection. By contrast, in the burst model, in the illustrated realization that leads to persistent infection (Figure 4), after the first virus infects a cell that cell lives about 1.25 days. No additional virus is produced until this cell dies and thus 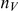 stays at zero until day 1.25 at which time a burst of virus is produced. While some of this newly produced virus infects new cells, the rest gets cleared and 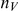 returns to zero until another cell dies at approximately 1.8 days. Additional cells are infected at this point and 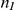 rises due to this and subsequent bursts of virus.

Realizations that lead to extinction are shown in the lower panels of Figures 3 and 4. Note the y-axes are scaled differently than in the cases that lead to persistent infection. In the continuous production case, by chance most of the produced virus is cleared and thus 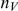 never gets above 3. Also, the number of infected cells remains small, reaching 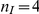, before these cells sequentially die and extinguish the infection. In the burst model, even though in the realization shown 10 virions are produced in each of three bursts, the first two bursts only lead to the infection of 1 cell each and virions in the last burst are all cleared without infecting any cells leading to the extinction of the infection.

Because a particular realization may not be representative of a stochastic process, we show in the left column of Figure 5 100 realizations of the continuous production model that lead to infection starting from a single virion, and in the right column 100 realizations that lead to extinction. Figure 6 is the same as Figure 5 except the initial condition is 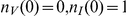, i.e., the infection is started by the introduction of a single infected cell. During sexual transmission of HIV it is not known whether infected cells or virus particles penetrate epithelial layers and initiate infection. For the burst model, Figures 7 and 8 show 100 realizations each of infection and clearance for the initial conditions (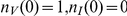) and (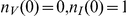) respectively. It can be seen that in none of the burst model realizations that lead to extinction were there ever more than a single infected cell. By contrast, the continuous model had several realizations in which 2 or 3 cells were infected but still went to extinction. Infected cells in the burst model always produce 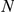 infectious virions (here 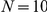). Infected cells in the continuous model realizations that led to extinction never produced more than 4 infectious virions total even though there were as many as 3 infected cells. The differences in the two models are fairly evident in the sets of realizations that lead to extinction. The differences in the realizations that lead to infection are not evident to the naked eye because the particle numbers start to get large and the models converge towards mean-field dynamics.

In a stochastic model each infection can have a different course and the scenarios described above even with 100 realizations need not be representative. We thus ran simulations until 100,000 realizations resulted in infection. For the continuous production model this occurred after a total of 429,639 simulations had been performed. Of these 429,639 simulations 329,639 resulted in extinction and 100,000 in infection. The resulting fraction of simulation that went extinct, 0.767, is in accord with our calculation of 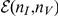. Note that for the continuous model 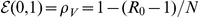. With 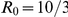 and 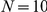 we find the extinction probability is 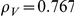.

For the burst model the single virion extinction probability, Eq. (18), gives 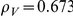. To check this, we performed simulations using the burst model until 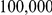 realizations resulted in infection. To achieve this a total of 306,592 simulations were performed. Of these 306,592 simulations, 206,592 resulted in extinction and 100,000 resulted in infection, yielding a 67.38% chance of extinction, in accord with the predicted value, 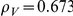. (The expected value for the number of extinctions in 306,592 Bernoulli trials is 206,336 and the standard deviation is 259.8.)

The analytical results that we have derived for the extinction probabilities do not provide any information about dynamics. Thus, the stochastic process could take hours, days or months before extinction is reached. To gain insight into these dynamics, we have plotted in Figures 9 and 10, for infections starting with a single virion, the fraction of simulations that go extinct at various times after infection, with Figure 9 for the continuous production model and Figure 10 for the burst model. These histograms represent the distributions of time to extinction conditioned on the process ultimately going extinct. Both the continuous production and burst models have a sharp initial decay in their conditional distributions of times to extinction. One might expect the extinction rate to be proportional to 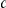, since that is the rate at which virions are cleared. However, from the graphs one can deduce that the initial decay occurs on a time scale given by 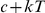. Since new cells are infected at rate 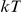 it is not completely self-evident that the initial decay should be given by 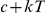.

The fact that the initial decay is given by 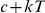 rather than just 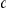 can be understood in terms of a simple 3-state Markov chain 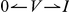, where the initial state is 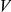, a single virion, i.e., 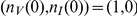, 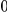 represents the extinction of the infecting virion, i.e. (0,0), and 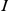 representing the virion infecting a new cell, i.e. (0,1). Given that extinction occurs, consider the conditional distribution of times for the system to make the transition from 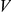 to 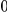. The probability that the system remains in state 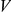 given that it was in state 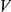 at time 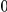, is just 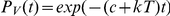 where 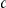 and 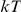 are the transition rates from 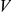 to 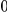 and 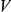 to 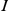, respectively. The probability flux from 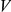 to 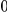 is just 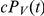. Let 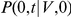 be the conditional probability that the system makes the transition into 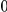 for the first time at time 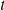, given that it was in state 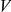 at time 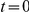. Then 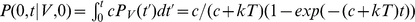. The conditional distribution of first passage times from 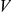 to 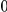 is then, 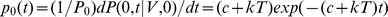, where 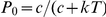 is the probability that the system transitioned into 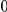 from 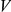. This is exactly analogous to the sharp initial decay with rate 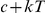 from the single virion initial condition.

After the initial transient, the distributions of times to extinction display long tails that decay roughly with rate 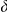. In both models the long tails are caused by the infection of cells. Once a cell is infected it takes much longer to reach extinction, on average, than before any cells are infected. The difference between the two models is largely due to the difference between the single infected cell extinction probability 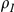, in the continuous and burst models, *i.e.*, 
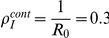
 and 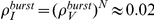 for our default parameter values. Extinction from an infected cell is much less likely for the burst model than for the continuous model. Thus there is substantially more probability in the tails (of the distribution of times to extinction starting from a single virion) for the continuous model than for the burst model. For the continuous model we have derived approximate analytic solutions to the full problem that we shall present elsewhere.

To further highlight the difference between the models, we examined the time needed to obtain a 95% probability of extinction given that the process goes extinct. For the default parameter values, the burst model reaches 95% (conditional) probability that the infection is extinct after about 2.5 hours, whereas the continuous model reaches this probability of extinction after about a half day. Thus, there is a significant difference in the behavior of systems governed by the continuous production and burst models. Note also that the conditional distribution of times for an arbitrary number of virions to go extinct can be inferred from the conditional single virion distribution of extinction times.

**Figure 11:**
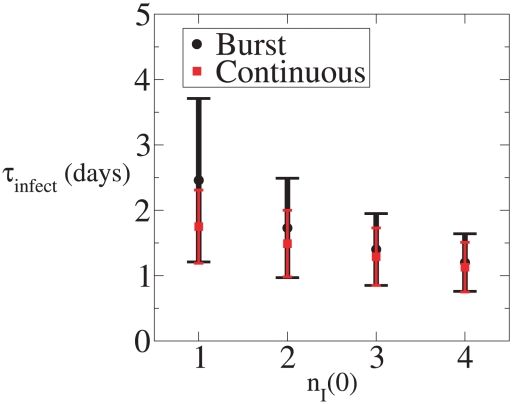
Mean time to reach 32 infected cells versus 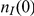. Black: Burst Model. Red: Continuous Production Model.

The time to extinction is difficult to determine experimentally, while the time to observable infection is not. Thus, we have studied the time it takes for infection to reach 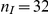, which as we have argued above is essentially the time for SIV to be detectable in a rhesus macaque, and which is also a measure of the time to reach a state comparable to established infection. For both the continuous and burst models we generated a 100,000 realizations in which 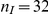 is reached. For these simulations, the distribution of times until 32 cells are infected is shown in Figure 9 and Figure 10 for the continuous production and burst models, respectively, and with infections initiated either with a single virion or with a single infected cell. The mean time to reach 32 infected cells in the burst model is 2.46 days and in the continuous production model 1.75 days for either initial condition. Here the two initial conditions give essentially the same result. In an infection started with a single virion, if the virion is cleared the process goes extinct. Since we have conditioned on this not occurring, the initiating virion must infect a cell, and hence it quickly generates the same state as initiating infection with a single infected cell. One also expects the burst and continuous models to converge to statistically indistinguishable behavior once the particle numbers are sufficiently high, well before there are 32 infected cells. The differences in the mean time to reach 32 infected cells starting from a single infected cell is substantial. This is because the early dynamics are dominated by stochastic effects. In Figure 11 we have plotted the mean time to infection from 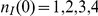 (and 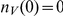) for the two models. For 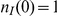 the differences are substantial but decrease with increasing 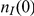.

## Discussion

The dynamics of acute HIV and SIV infection have been modeled deterministically by a number of authors [32], [33], [41]–[43], and in some cases these models have been used to fit data and extract best-fit parameter values. However, despite the success of these models they do not properly capture the very earliest dynamics of infection where stochastic effects may play a large role. Recent data has convincingly established that a large fraction of infections are established by one or a few infectious virions or infected cells [4], [44]–[48]. If during sexual transmission only a few virions or infected cells are actually transmitted from one infected person to another then one would expect that a large fraction of sexual encounters between an infected and uninfected person might not lead to successful viral transmission. Epidemiological studies support this and have concluded that HIV transmission occurs at frequencies of between 1 in 100 and 1 in 1,000 coital acts [3]. Similarly, experimental studies of SIV infection by intrarectal inoculation of virus has shown that at low doses not every encounter with virus leads to detectable infection and that there is substantial variability in the number of inoculations needed to establish detectable infection [47]. Further, as with HIV when infection was detectable, in most cases it appeared that only one or a few viral genomes established the infection. Lastly, one study aimed at detecting HIV-1 at the earliest possible moments in infection using a qualitative assay that could detect the presence of 4 HIV-1 RNA copies/ml with 95% accuracy showed that in some individuals a period of intermittent low-level viremia preceded the period of steadily rising viremia previously studied with deterministic models [49]. Intermittent low level viremia and frequent extinction of infection is precisely what would be expected by a stochastic model as shown by our stochastic stimulations.

A number of previous authors have also performed stochastic simulations of HIV infection [10]–[14], [18], [50]. What is novel here is that we have shown that the stochastic extinction probability, 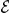, for early infection models is amenable to exact solution under the assumption that clearance of each infecting virion and infecting cell occurs independently. We validated the predictions of this analysis via stochastic simulations based on the standard model of viral infection. That our model and simulations agree is not surprising as in the basic target-cell limited model each virion and infected cell acts independently. One can think of situations where this does not hold; for example, if a threshold number of infected cells is required to generate an immune response that then rapidly clears the infection. Thus, while mathematically it is fairly clear when the independence assumption holds, and most current models of early HIV dynamics that ignore immune responses are consistent with this assumption, whether real viral extinction processes are in fact independent is an experimental question. There is at least one report of experiments on rhesus macaques in which it appears that repeated low dose challenges are cleared independently, suggesting that immune responses are not generated during exposures that lead to viral extinction as assumed by our models [51].

Although we have not done so here, one can use our analytical results on extinction probabilities to explore the parameter ranges that give rise to different probabilities of extinction. For example, if one assumes that extinction occurs 99% of the time so as to yield a 1% chance of infection in a coital act, in which say 1 infectious virion is transmitted to an uninfected individual, then one requires that 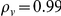. Then for the continuous production model, Eq. (15), predicts that with 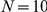 one requires 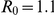, and with 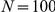 one requires 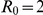. While values of 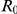 in the literature are higher than this [32]–[34] they were obtained from viral load measurements obtained after the viral level has reached 50 HIV RNA copies/ml or higher. Thus, very early in infection 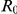 may be much smaller than determined later in infection or 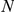 may be larger than assumed here. Experimental validation of these possibilities is needed.

To further explore potential parameter ranges, Chen et al. [35] estimate that in SIV infection 50,000 virions can be released from an infected cell. Further, Ma et al. [40] showed that when 10 SIV particles taken from a recently infected macaque were injected intravenously into two other macaques, both became infected, indicating that the ratio of infectious particles to virions was between 0.1 and 1 in this experiment. To see if these numbers make sense in the context of our extinction calculation, assume that of the 50,000 virions released 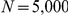 were infectious. Also, assume there is only a 0.1% chance of infection per coital act as frequently cited for stable couples with low prevalence of high-risk cofactors [3]. Then by Eq. (15) with 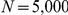, we find 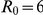, which is in the range estimated by Stafford et al. [33] and Ribeiro et al. [32] for acute HIV infection. This example shows that various parameter estimates in the recent literature are consistent with the findings of our model. However, the fact that the two monkeys injected intravenously with 10 SIV particles became infected is not consistent with the 0.1% infection rate per coital act assumed above. Clearly, sexual transmission and direct injection of virus into the blood stream are very different events. Further, if 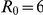 and 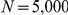 infectious particles, then from the definition of 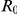, Eq, (5), 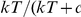) = 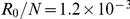, and an estimate of 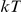 can be made if a value of 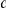 is assumed. In our simulations we used 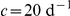 which yields 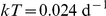 (for infectious virions), but higher values of 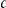 are possible depending upon whether one is estimating clearance from blood or lymphoid tissue as recently discussed by De Boer et al. [52]. Clearly, direct measurements of these parameters during acute infection still needs to be done, but these example provide some guidelines as to what we might expect.

Our calculations focused on determining 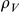 and 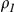, the probabilities of an infection starting from one virion or from one infected cell going extinct, respectively. Once these probabilities are determined it is straightforward to analyze circumstances where more than one virion or one infected cell initiates infection. For example, assume that 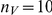 infectious virions are transmitted to a recipient and initiates infection. Frequently only one viral genome is identified by sequencing [4]. One explanation for this observation is that 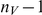 of the virions lead to extinction and only one virion founds a successful infection. If we assume that successful infection only occurs in 1 per 1,000 coital acts [3], then 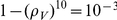 or 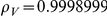. Further, the probability of only one viral genome founding the infection, given that infection occurs, is given by the conditional binomial distribution, i.e., 
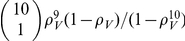
, which with 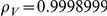, occurs with probability 0.9995. Thus, even if 10 infectious virions are transmitted, if successful infection is rare, as in this example, one is almost assured that only one virus will grow and found the infection.

Another unique aspect of our work is that we show in a stochastic setting continuous viral production can be distinguished from viral production that occurs in a burst. In at least one lentivirus, visna virus, the greatest fraction of virus production occurs towards the end of the viral life cycle [21], more consistent with a burst model than a model with constant continuous production. For HIV it has not yet been established whether a burst or continuous production model is most appropriate. One might envision viral production from a highly activated CD4+ T cell to occur in a process approximating a burst, whereas production from an infected resting CD4+ T cell or from an infected macrophage, where infected cell life spans might be weeks rather than days [53], might be continuous. In simple deterministic models, such as the standard model of viral infection, burst versus continuous production can not be distinguished, and give rise to identical dynamics. Here we show that the probability of extinction is different for continuous production and burst production and that the time to establish infection differs between these two modes of production.

Our core result is that with the burst model one obtains lower extinction probabilities (see Figure 2) and longer times to the establishment of infection than with the continuous production model (see Figures 9, 10 and 11), even when the mean number of virions produced is the same. In the continuous production model virus production starts as soon as a cell is infected and these released virions can infect other cells leading to a more rapid establishment of infection than with the burst model. Further, with continuous virion production there is more heterogeneity in the number of virions an infected cell produces owing to the variability in infected cell lifespans. In fact, there is a chance an infected cell will die before producing any virions. This in turn leads to a greater chance of the process going extinct. In epidemic models a similar effect has been noted, where for 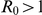, increased variability in individual infectiousness increases the probability of stochastic extinction [54].

In the continuous production model we have assumed that the rate of virion production is constant. In prior work using deterministic models to describe HIV dynamics, more realistic models of viral production have been studied in which the rate of viral production varies continuously over the cell's lifespan [55]–[57]. In such models the rate of viral production is described by a function 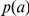, where 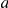 denotes the age or length of time a cell has been infected. Our continuous production and burst model are two choices of possible functions, i.e. 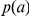 = constant and 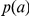 being a Dirac delta function. Clearly many other choices are possible. Such age-structured HIV production models have not yet been analyzed in a stochastic context.

In the burst model we first assumed that each cell produces exactly 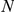 virions. As this is unlikely to be true, we then generalized this by allowing 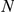 to be a random variable. Viral production at the individual cell level still remains to be measured and thus nothing is known about in vivo burst size distributions. Further, in both cases the burst size was not coupled to the cell's lifespan. Another possible extension of our model is to allow the lifespan of a cell to be influenced by the rate of viral production or the viral burst size. Cells that use resources to produce virus rapidly might die sooner. Alternatively, one could envision that the amount of virus produced by a cell is influenced by the cell's lifespan. For example, if a cell produces virus at a constant rate and then releases it in a burst, then a cell that lives longer would have the opportunity to make more virus. Couplings between cell lifespan and viral production have been studied previously in deterministic models by a number of authors [55]–[58].

Because our model is derived from the standard model of viral infection it carries over features and limitations of that model. In particular, both the standard model and our continuous production model assume that once a cell is infected it begins producing virus immediately. Also, in the burst model even if a cell lives an infinitesimal amount of time after being infected it releases a full burst of virus. In reality, many steps of the viral life cycle need to be completed before viral production can occur. It is straight forward to refine our models so that infected cells wait a period of time before they can begin to produce virus. This has been done previously in the context of differential equation models [59], [60]. Obviously, in the stochastic model the waiting time distributions until extinction or infection are strongly affected by such a modification. On the other hand, the extinction probabilities remain unaltered if one assumes infected cells before they begin to produce virus have negligible death rates. Including death of such cells will require a modification of the extinction probability calculations.

The analysis we have presented assumes that target cell levels remain constant. This assumption is valid early in infection if we assume the system is well-mixed as there are approximately 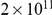 CD4^+^ T cells in a human [61], and perhaps 10-fold less in a macaque, and our model only follows the infection process until 32 cells are infected. At longer times, once the number of infected cells get large enough to have an impact on target cell numbers, stochastic fluctuations would be of no significance in the context of a well-mixed system and deterministic models should be appropriate. If HIV is introduced into the blood, say through transfusion or by intravenous drug use, then the well-mixed assumption with no target cell limitation would seem appropriate. However, in sexual transmission, one could envision that spatial effects near the site of transmission are important and in the region that the entering virions or infected cells find themselves in target cells maybe rare and hence limiting. Thus, it might be of interest to study the nonlinear problem in which target cell numbers vary. One could also envision situations in which immune responses are included in the model, such as in studies of vaccine-induced protection, and in which stochastic effects are important in describing the early immune response. Such extensions of our model remain to be developed.

Lastly, our model has not yet addressed the question of how the initial infecting agents, infectious virions or infected cells, get access to target cells. In experiments involving intrarectal or intravaginal challenge of rhesus macaques large numbers of virions have been introduced, e.g. 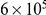 to 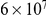 in the experiments by Keele et al. [47]. Nonetheless, only one or a few viral genomes were seen to expand in infected animals. Whether larger numbers cross epithelial barriers and are then rapidly eliminated or whether the barrier itself prevents all but a few viral genomes to gain access to target cells and expand is not known, Thus, models and further experiments examining these early steps are still required.

In conclusion, we have developed stochastic models of early viral infection in which continuous viral production and burst viral production are distinguished. The models capture the stochastic aspects of some of the earliest events in infection and provide quantitative insights into the possibility that early infection will go extinct rather than become established. We provide analytical solutions for the extinction probability and, via simulation, insights into the distribution of times until infection goes extinct or becomes established.

## References

[1] Gray R, Wawer M, Brookmeyer R, Sewankambo N, Serwadda D (2001). Probability of HIV-1 transmission per coital act in monogamous, heterosexual, HIV-1-discordant couples in Rakai, Uganda.. Lancet.

[2] Wawer M, Gray R, Sewankambo N, Serwadda D, Li X (2005). Rates of HIV-1 transmission per coital act, by stage of HIV-1 infection, in Rakai, Uganda.. J Infect Dis.

[3] Powers KA, Poole C, Pettifor AE, Cohen MS (2008). Rethinking the heterosexual infectivity of HIV-1: a systematic review and meta-analysis.. Lancet Infect Dis.

[4] Keele BF, Giorgi EE, Salazar-Gonzalez JF, Decker JM, Pham KT (2008). Identification and characterization of transmitted and early founder virus envelopes in primary HIV-1 infection.. Proc Natl Acad Sci USA.

[5] Li Q, Skinner PJ, Ha SJ, Duan L, Mattila TL (2009). Visualizing antigen-specific and infected cells in situ predicts outcomes in early viral infection.. Science.

[6] Feller W (1968). An Introduction to Probability Theory and Its Applications, Vol. 1.

[7] Edwards A (1983). Pascal's problem - the gambler's ruin.. Int Statistial Review.

[8] Huygens C (1714). Christiani Hugenii libellus de ratiociniis in ludo ale. Or, the value of all chances in games of fortune; … mathematically demonstrated. London: printed by S. Keimer for T. Woodward..

[9] Tan WY, Wu H (1998). Stochastic modeling of the dynamics of CD4+ T-cell infection by HIV and some Monte Carlo studies.. Math Biosci.

[10] Kamina A, Makuch RW, Zhao H (2001). A stochastic modeling of early HIV-1 population dynamics.. Math Biosci.

[11] Heffernan J, Wahl L (2005). Monte Carlo estimates of natural variation in HIV infection.. J Theoret Biol.

[12] Tuckwell HC, Corfec EL (1998). A stochastic model for early HIV-1 population dynamics.. J Theoret Biol.

[13] Corfec EL, Tuckwell HC (1998). Variability in early HIV-1 population dynamics.. AIDS.

[14] Merrill SJ (2005). The stochastic dance of early HIV infection.. J Comput Appl Math.

[15] Lee H, Giorgi E, Keele B, Gashen B, Athreya G (2009). Modeling sequence evolution in acute HIV-1 infection.. J Theoret Biol.

[16] Tuckwell HC, Shipman PD, Perelson AS (2008). The probability of HIV infection in a new host and its reduction with microbicides.. Math Biosci.

[17] Haeno H, Iwasa Y (2007). Probability of resistance evolution for exponentially growing virus in the host.. J Theor Biol.

[18] Ribeiro RM, Bonhoeffer S (2000). Production of resistant HIV mutants during antiretroviral therapy.. Proc Natl Acad Sci USA.

[19] Anderson RM, May RM (1991). Infectious Diseases of Humans: Dynamics and Control.

[20] Perelson AS (2002). Modelling viral and immune system dynamics.. Nat Rev Immunol.

[21] Haase A, Stowring L, Harris J, Traynor B, Ventura P (1982). Visna DNA synthesis and the tempo of infection in vitro.. Virology.

[22] Perelson A, Nelson P (1999). Mathematical analysis of HIV-1 dynamics in vivo.. SIAM Rev.

[23] Nowak MA, May RM (2000). Virus Dynamics: Mathematical Principles of Immunology and Virology.

[24] Gillespie DT (1977). Exact stochastic simulation of coupled chemical reactions.. J Phys Chem.

[25] Redner S (2001). A Guide to First-Passage Processes.

[26] Parzen E (1960). Modern Probability Theory and Its Applications.

[27] Mosteller F (1987). Fifty Challenging Problems in Probability with Solutions.

[28] Britton T, Lindenstrand D (2009). Epidemic modelling: Aspects where stochasticity matters.. Math Biosci.

[29] Britton T (2010). Stochastic epidemic models: A survey.. Math Biosci.

[30] Markowitz M, Louie M, Hurley A, Sun E, Mascio MD (2003). A novel antiviral intervention results in more accurate assessment of human immunodeficiency virus type 1 replication dynamics and t-cell decay in vivo.. J Virol.

[31] Ramratnam B, Bonhoeffer S, Binley J, Hurley A, Zhang L (1999). Rapid production and clearance of HIV-1 and hepatitis C virus assessed by large volume plasma apheresis.. Lancet.

[32] Ribeiro RM, Qin L, Chavez LL, Dongfen L, Self SG (2010). Estimation of the initial viral growth rate and the basic reproductive number during acute HIV-1 infection.. J Virol.

[33] Stafford MA, Corey L, Cao Y, Daar E, Ho DD (2000). Modeling plasma virus concentration during primary HIV infection.. J Theoret Biol.

[34] Little SJ, McLean AR, Spina CA, Richman DD, Havlir DV (1999). Viral dynamics of acute HIV-1 infection.. J Exp Med.

[35] Chen HY, Mascio MD, Perelson AS, Ho DD, Zhang L (2007). Determination of virus burst size in vivo using a single-cycle SIV in rhesus macaques.. Proc Natl Acad Sci USA.

[36] Bourinbaiar AS (1994). The ratio of defective HIV-1 particles to replication-competent infectious virions.. Acta Virologica.

[37] Kwon Y, Hung G, Anderson W, Peng C, Yu H (2003). Determination of infectious retrovirus concentration from colony-forming assay with quantitative analysis.. J Virol.

[38] Marozsan A, Fraundorf E, Abraha A, Baird H, Moore D (2004). Relationships between infectious titer, capsid protein levels, and reverse transcriptase activities of diverse human immunodeficiency virus type 1 isolates.. J Virol.

[39] Rusert P, Fischer M, Joos B, Leemann C, Kuster H (2004). Quantification of infectious HIV-1 plasma viral load using a boosted in vitro infection protocol.. Virology.

[40] Ma ZM, Stone MMP, Schweighardt B, Haigwood NL (2009). High specific infectivity of plasma virus from the pre-ramp-up and ramp-up stages of acute simian immunodeficiency virus infection.. J Virol.

[41] Phillips A (1996). Reduction of HIV concentration during acute infection: Independence from a specific immune response.. Science.

[42] Nowak MA, Lloyd AL, Vasquez GM, Wiltrout TA, Wahl LM (1997). Viral dynamics of primary viremia and antiretroviral therapy in simian immunodeficiency virus infection.. J Virol.

[43] Vaidya NK, Ribeiro RM, Miller CJ, Perelson AS (2010). Viral dynamics during primary SIV infection: Effect of time-dependent virus infectiousness.. J Virol.

[44] Abrahams M, Anderson J, Giorgi E, Seoighe C, Mlisana K (2009). Quantitating the multiplicity of infection with human immunodeficiency virus type 1 subtype c reveals a non-Poisson distribution of transmitted variants.. J Virol.

[45] Haaland RE, Hawkins PA, Salazar-Gonzalez J, Johnson A, Tichacek A (2009). Inammatory genital infections mitigate a severe genetic bottleneck in heterosexual transmission of subtype A and C HIV-1.. PLoS Pathogens.

[46] Kearney M, Maldarelli F, Shao W, Margolick JB, Daar ES (2009). Human immunodeficiency virus type 1 population genetics and adaptation in newly infected individuals.. J Virol.

[47] Keele BF, Li H, Learn GH, Hraber P, Giorgi EE (2009). Low-dose rectal inoculation of rhesus macaques by SIVsmE660 or SIVmac251 recapitulates human mucosal infection by HIV-1.. J Exp Med.

[48] Fischer W, Ganusov V, Giorgi E, Hraber P, Leitner T (2010). Transmission of single HIV-1 genomes and dynamics of early immune escape revealed by ultra-deep sequencing.. PLoS One.

[49] Fiebig EW, Heldebrant CM, Smith RIF, Conrad AJ, Delwart EL (2005). Intermittent low-level viremia in very early primary HIV-1 infection.. JAIDS.

[50] Merrill SJ (1989). Modeling the interaction of HIV with cells of the immune system.

[51] Letvin NL, Rao SS, Dang V, Buzby AP, Korioth-Schmitz B (2007). No evidence for consistent virus-specific immunity in simian immunodeficiency virus-exposed, uninfected rhesus monkeys.. J Virol.

[52] Boer RJD, Ribeiro RM, Perelson AS (2010). Current estimates for HIV-1 production imply rapid viral clearance in lymphoid tissue.. PLoS Computational Biol.

[53] Perelson A, Essunger P, Cao Y, Vesanen M, Hurley A (1997). Decay characteristics of HIV-1-infected compartments during combination therapy.. Nature.

[54] Lloyd-Smith JO, Schreiber SJ, Kopp PE, Getz WM (2005). Superspreading and the effect of individual variation on disease emergence.. Nature.

[55] Coombs D, Gilchrist MA, Percus J, Perelson AS (2003). Optimal viral production.. Bull Math Biol.

[56] Nelson PW, Gilchrist MA, Coombs D, Hyman JM, Perelson AS (2004). An age-structured model of HIV infection that allows for variations in the production rate of viral particles and the death rate of productively infected cells.. Math Biosci Engin.

[57] Gilchrist M, Coombs D, Perelson A (2004). Optimizing within-host viral fitness: infected cell lifespan and virion production rate.. J Theoret Biol.

[58] Komarova NL (2007). Viral reproductive strategies; how can lytic viruses be evolutionarily competitive?. J Theoret Biol.

[59] Herz AVM, Bonhoeffer S, Anderson RM, May RM, Nowak MA (1996). Viral dynamics in vivo: Limitations on estimates of intracellular delay and virus decay.. Proc Natl Acad Sciences USA.

[60] Mittler J, Sulzer B, Neumann A, Perelson A (1998). Inuence of delayed viral production on viral dynamics in HIV-1 infected patients.. Math Biosci.

[61] Zhang ZQ, Notermans DW, Sedgewick G, Cavert W, Wietgrefe S (1998). Kinetics of CD4+ T cell repopulation of lymphoid tissues after treatment of HIV-1 infection.. Proc Natl Acad Sci USA.

